# Supplementary material for: KNN-based frequency-adjustable ferroelectric heterojunction and biomedical applications
Source: Nat Commun. 2025 Aug 2;16:7120. doi: 10.1038/s41467-025-62079-0 (PMC12317977; doi:10.1038/s41467-025-62079-0)
Supplement: Supplementary file 1 — Supplementary Information [file 41467_2025_62079_MOESM1_ESM.pdf]

Supplementary Information for

KNN-based frequency-adjustable ferroelectric heterojunction and biomedical applications

Tao Zhang[1,†], Haoyuan Hu[2,3,†], Hong Jiang[2,3,*], Zhen Wang[4], Jinfeng Lin[5], Ye Cheng[2,3], Wei Guo[2,3], Di Ke[1], Hai Hang[1], Mengshu Ta[1], Jun Ou-Yang[1], Jiwei Zhai[5], Xiaofei Yang[1], Songyun Wang[2,3,*], Benpeng Zhu[1,*]

[1] School of Integrated Circuit, Huazhong University of Science and Technology; Wuhan, 430074, China.

[2] Cardiovascular Hospital, Renmin Hospital of Wuhan University; Wuhan, 430061, China.

[3] Cardiac Autonomic Nervous System Research Center, Wuhan University; Wuhan, 430061, China.

[4] National Institute of Dental and Craniofacial Research (NIDCR), National Institutes of Health (NIH); Bethesda, MD 20892, USA.

[5] School of Materials Science and Engineering, Tongji University; Shanghai, 201804, China.

[†] These authors contributed equally: Tao Zhang, Haoyuan Hu.

[*] Corresponding author. Hong Jiang: hong-jiang@whu.edu.cn; Songyun Wang: wsy7982@126.com; Benpeng Zhu: benpengzhu@hust.edu.cn

The PDF file includes:

Supplementary Figs. 1 to 24
Supplementary Tables 1 to 2

Contents

Supplementary Figures

Supplementary Fig. 1. Entropy modulation strategy and element analysis based on electron probe microanalyzer (EPMA) of the KNN-3.5BHT ceramic.

Supplementary Fig. 2. Raman shift and piezoelectric properties of KNN ceramics.

Supplementary Fig. 3. The size of KNN piezoelectric composites.

Supplementary Fig. 4. Structural analysis of the f-FH.

Supplementary Fig. 5. The size and mass measurement of the f-FH device.

Supplementary Fig. 6. Transcranial ultrasound testing of the f-FH.

Supplementary Fig. 7. Transcranial focused sound pressure output of the f-FH.

Supplementary Fig. 8. The representative photos of stimulation.

Supplementary Fig. 9. Representative brain immunofluorescence scanning of the PVN.

Supplementary Fig. 10. f-FH stimulation of the PVN suppresses microglial activation and P2X7R expression.

Supplementary Fig. 11. f-FH stimulation to the PVN suppresses astrocyte activation and P2X7R expression.

Supplementary Fig. 12. Expression level of serum (a) NE and (b) NPY (n = 6).

Supplementary Fig. 13. Heart rate variability (HRV) changes after stimulation.

Supplementary Fig. 14. Typical TEM images of infarcted myocardium.

Supplementary Fig. 15. Analysis of neovascularization in myocardium.

Supplementary Fig. 16. Representative electrocardiograms of ventricular arrhythmia induced by protocol electrophysiological stimulation.

Supplementary Fig. 17. The cardiac function parameters in the three groups.

Supplementary Fig. 18. The heart specimens in the control, MI, and MI+f-FH groups.

Supplementary Fig. 19. Temperature changes in surrounding tissue of the PVN induced by the f-FH.

Supplementary Fig. 20. The behavioral observation following the implantation of the f-FH over time.

Supplementary Fig. 21. Representative HE staining of skin tissue in the control, MI,

and MI+f-FH groups.

Supplementary Fig. 22. Histological examination of major organs following the PVN ultrasound stimulation.

Supplementary Fig. 23. Statistical analysis of blood routine parameters in three groups (n = 3, respectively).

Supplementary Fig. 24. The changes of liver and kidney function in three groups (n = 3, respectively).

Supplementary Tables

Supplementary Table 1. Parameters of KNN piezoelectric composites.

Supplementary Table 2. Parameters of the f-FH stimulation for the MI treatment.

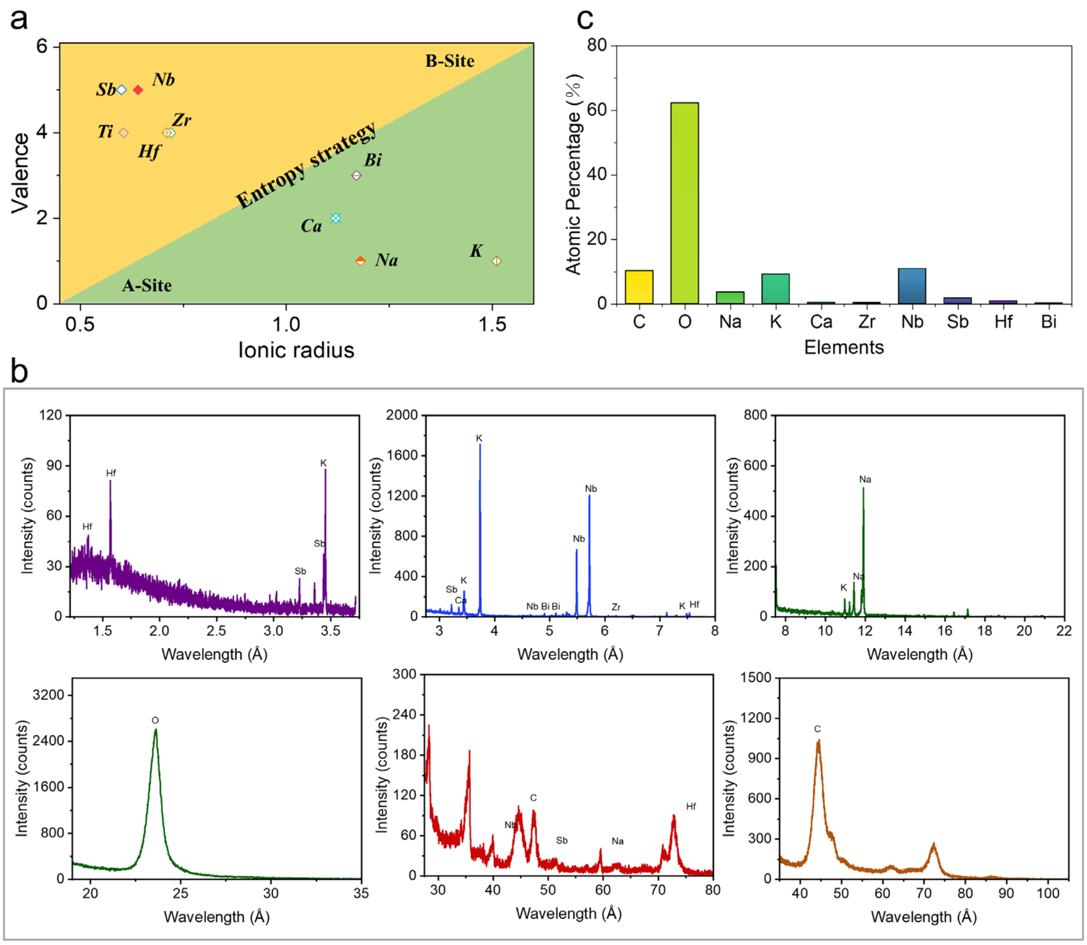

Supplementary Fig. 1. Entropy modulation strategy and element analysis based on electron probe microanalyzer (EPMA) of the KNN-3.5BHT ceramic. a The design strategy of medium-entropy for high-performance KNN-based piezoceramics. **b** Quantitative elemental analysis using wavelength-dispersive spectrometers (WDS), such as C, O, K, Ca, Na, Nb, Sb, Zr, Hf, and Bi. **c** Atomic percentage of microregions based on EPMA of the KNN-3.5BHT ceramic.

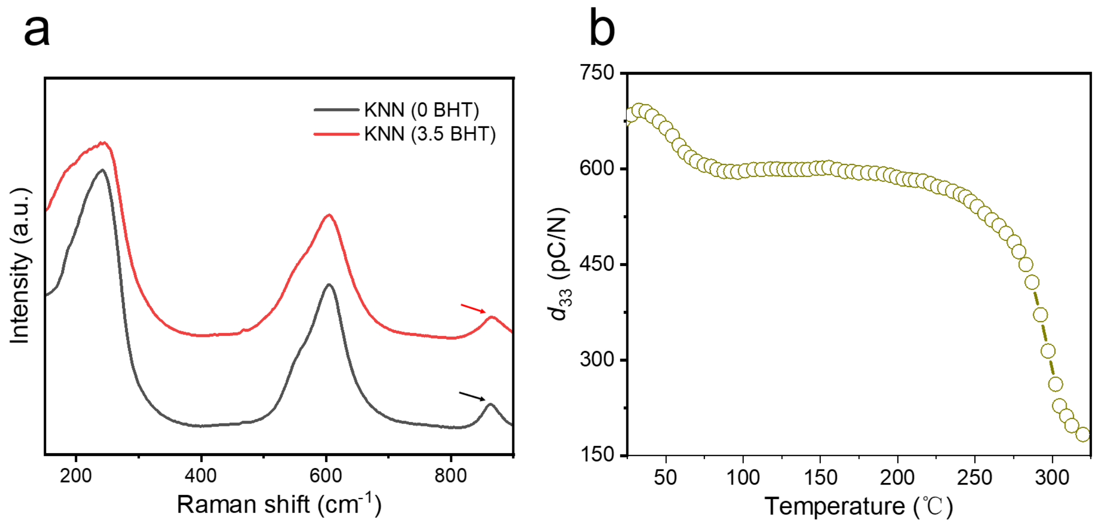

Supplementary Fig. 2. Raman shift and piezoelectric properties of KNN ceramics. (a) Raman shift of the piezoelectric ceramics (KNN and KNN-3.5BHT). (b) Temperature fatigue resistance of piezoelectricity (KNN-3.5BHT).

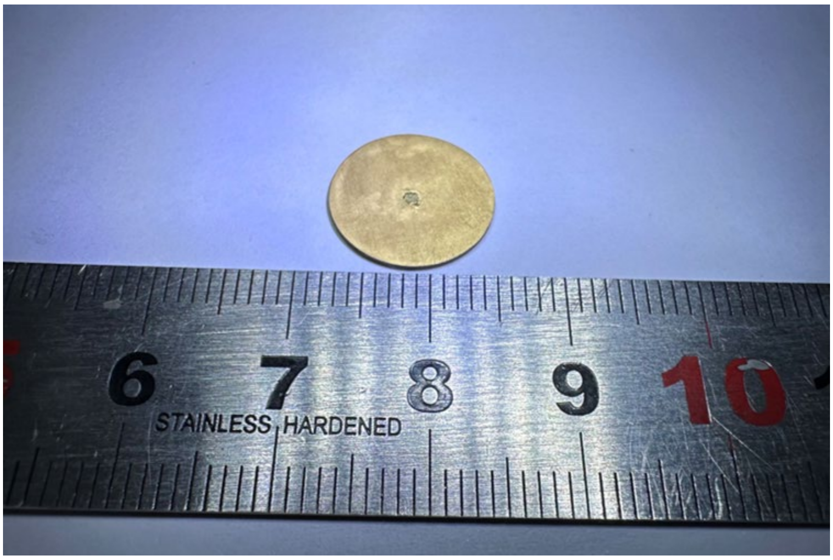

Supplementary Fig. 3. The size of KNN piezoelectric composites.

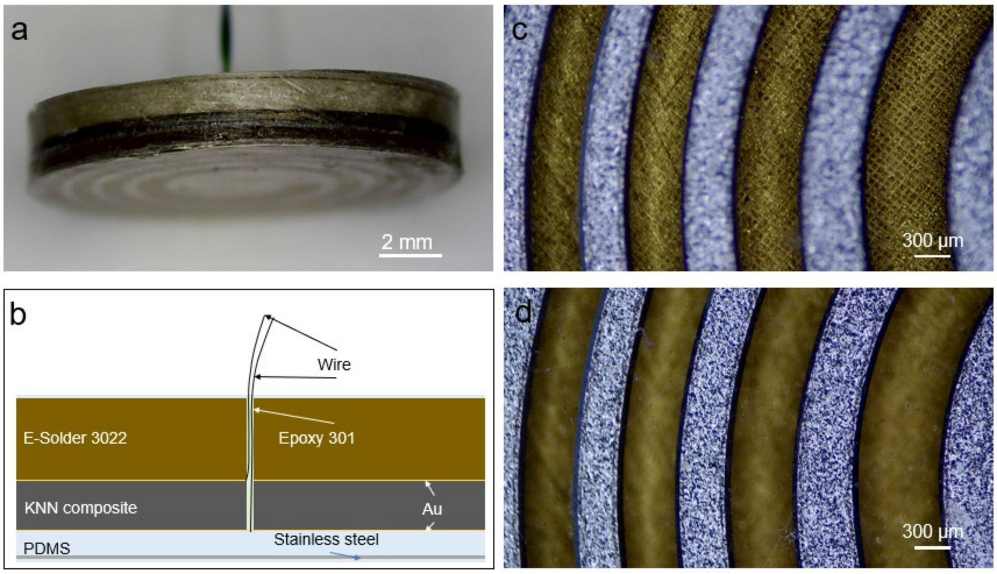

Supplementary Fig. 4. Structural analysis of the f-FH. a Side view of the device. **b** Cross section diagram of the device structure. **c** Detailed view of the composite within the device. **d** Enlarged view of the stainless-steel based Fresnel spiral plate inside the device.

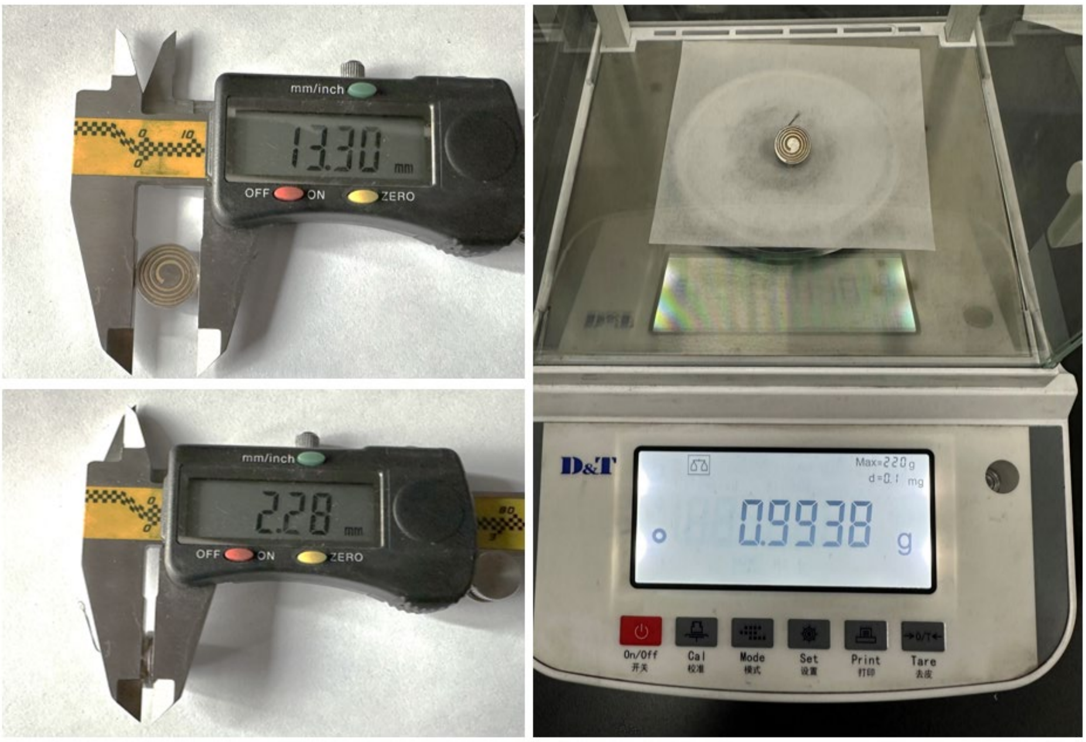

Supplementary Fig. 5. The size and mass measurement of the f-FH device.

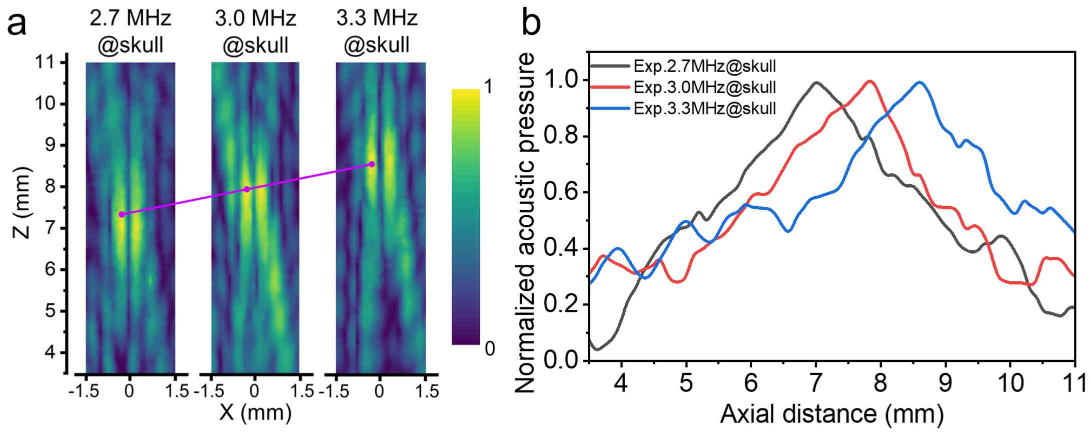

Supplementary Fig. 6. Transcranial ultrasound testing of the f-FH. a The acoustic pressure on the y = 0 plane radiated by the f-FH, at $f = 2.7$, 3.0, 3.3 MHz with skull. **b** Normalized axial pressure distribution.

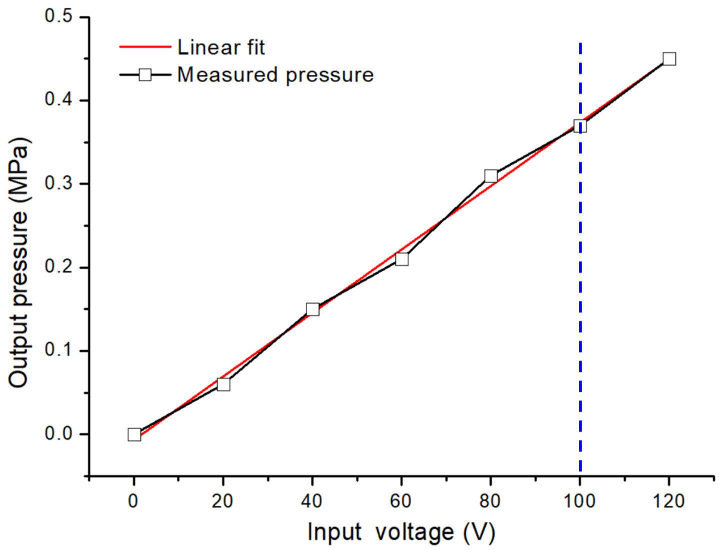

Supplementary Fig. 7. Transcranial focused sound pressure output of the f-FH.

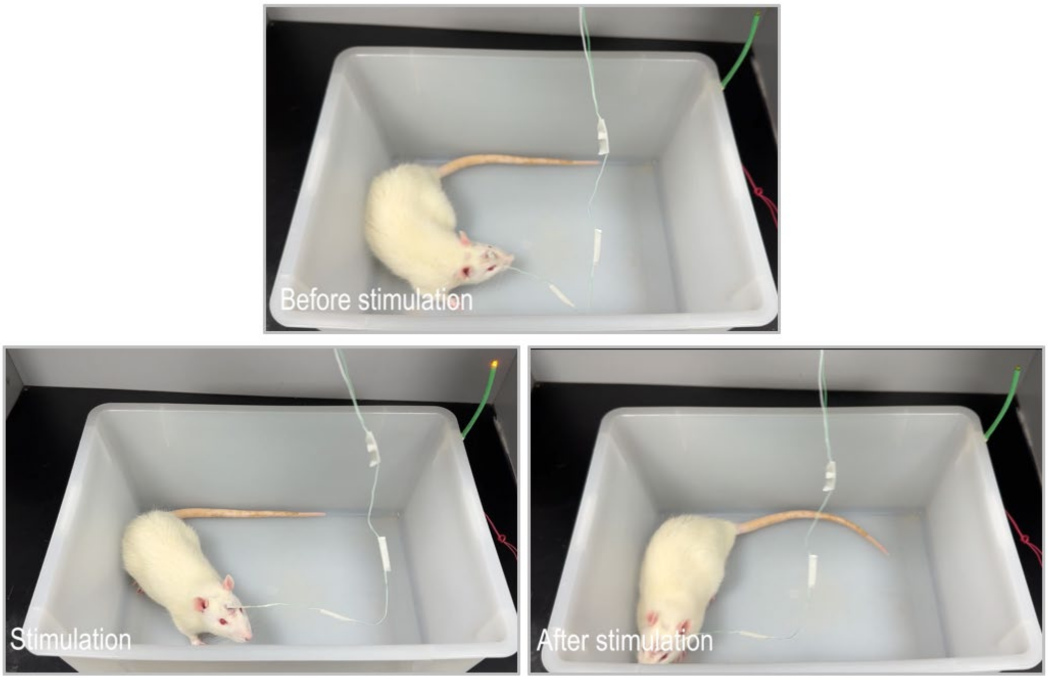

Supplementary Fig. 8. The representative photos of stimulation.

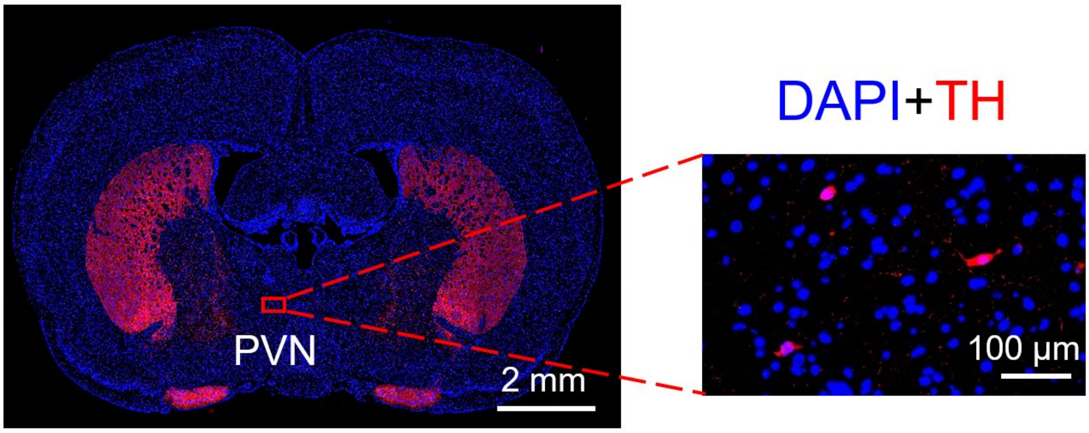

Supplementary Fig. 9. Representative brain immunofluorescence scanning of the PVN.

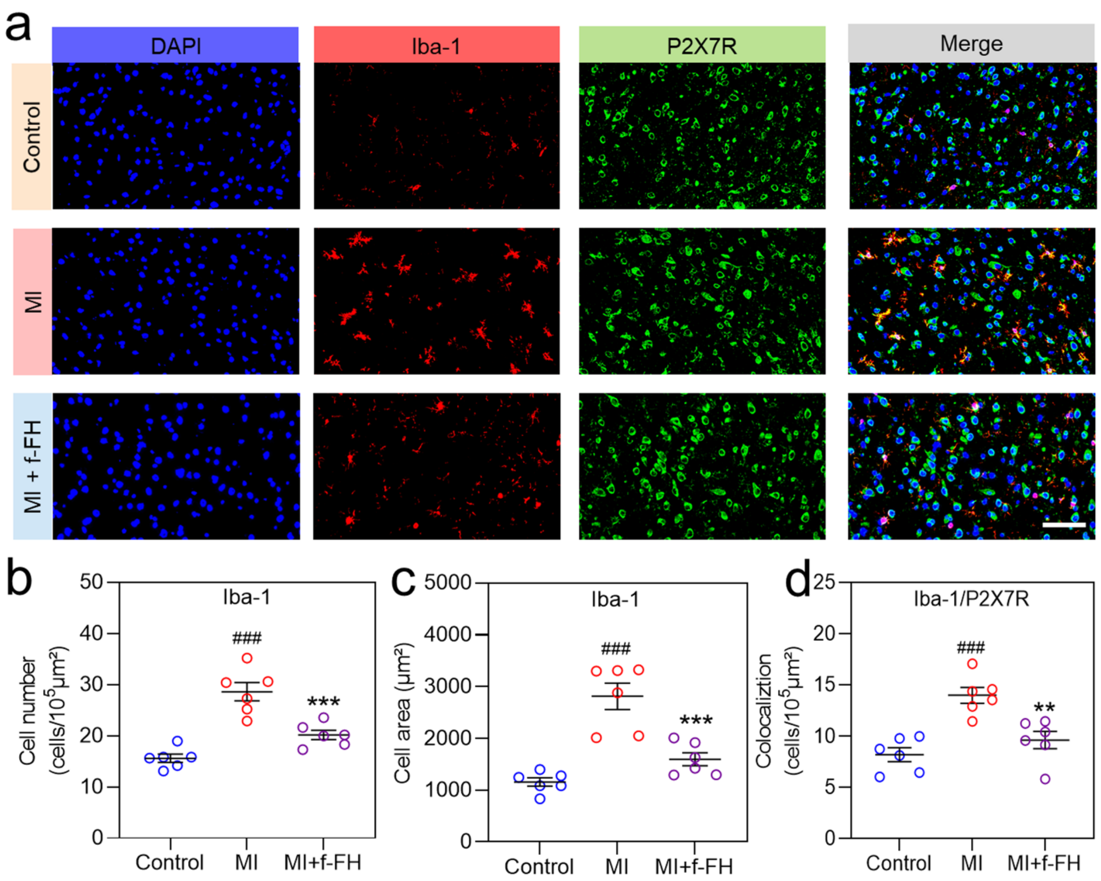

Supplementary Fig. 10. f-FH stimulation of the PVN suppresses microglial activation and P2X7R expression. a Representative images of immunofluorescence double staining for Iba-1/P2X7R in the PVN. Scale bar = 50 μm. Quantitative analysis of the number (**b**) and area (**c**) of Iba-1+ cells. **d** Quantitative analysis of the number of Iba-1+/P2X7R+ cells. Compared to the control group, $^{###}p < 0.001$; compared to the MI group, $^{**}p < 0.01$, $^{***}p < 0.001$.

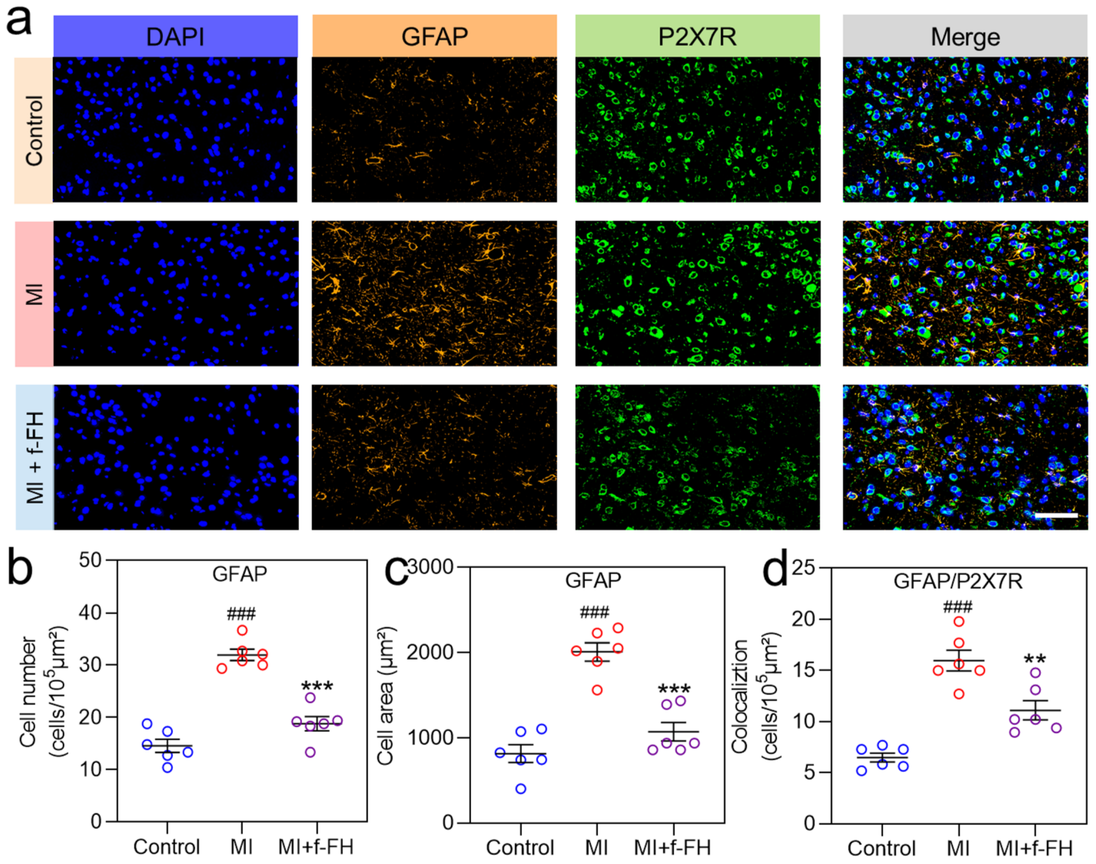

Supplementary Fig. 11. f-FH stimulation to the PVN suppresses astrocyte activation and P2X7R expression. a Representative images of immunofluorescence double staining for GFAP/P2X7R in the PVN. Scale bar = 50 μm. Quantitative analysis of the number (**b**) and area (**c**) of GFAP+ cells. **d** Quantitative analysis of the number of GFAP+/P2X7R+ cells. Compared to the control group, $^{###}p < 0.001$; compared to the MI group, $^{**}p < 0.01$, $^{***}p < 0.001$.

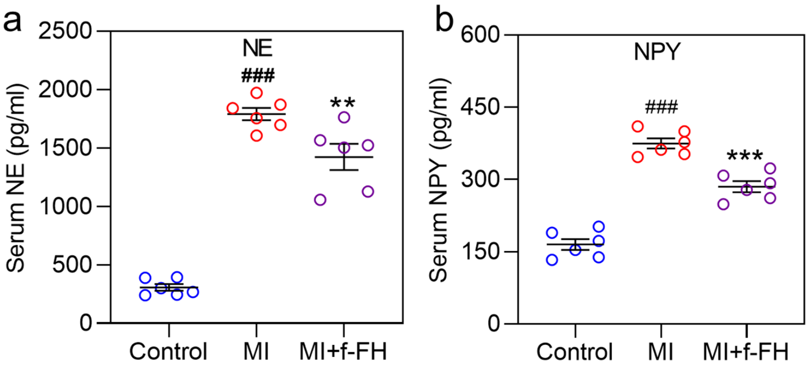

Supplementary Fig. 12. Expression level of serum (a) NE and (b) NPY (n = 6). Compared to the control group, $^{###}p < 0.001$; compared to the MI group, $^{**}p < 0.01$, $^{***}p < 0.001$.

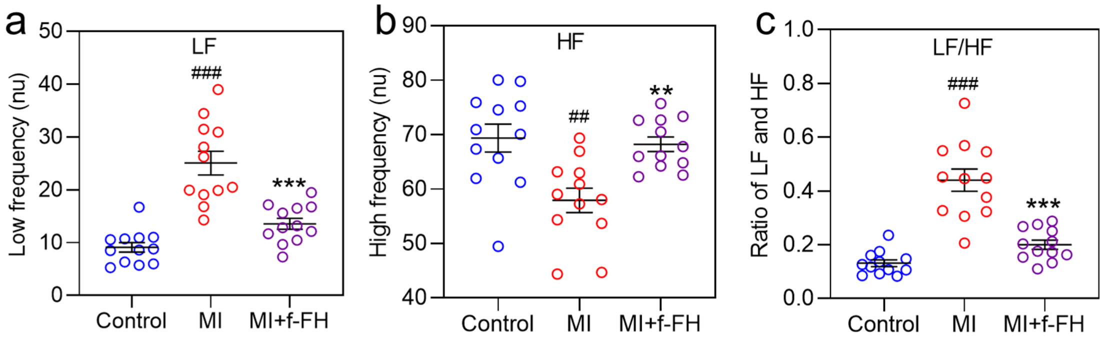

Supplementary Fig. 13. Heart rate variability (HRV) changes after stimulation. a LF reflecting sympathetic tone. **b** HF indicating parasympathetic tone, and (**c**) The ratio of LF and HF showing the relative activity of the sympathetic/parasympathetic nervous system ($n = 12$). Compared to the control group, $^{\#\#}p < 0.01$, $^{\#\#\#}p < 0.001$; compared to the MI group, $^{**}p < 0.01$, $^{***}p < 0.001$.

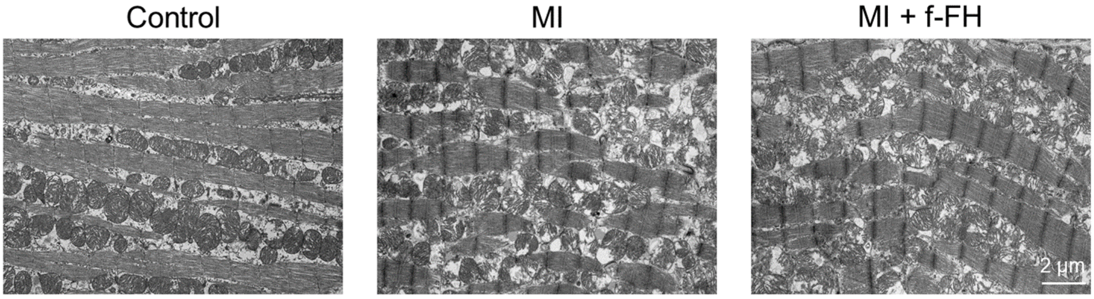

Supplementary Fig. 14. Typical TEM images of infarcted myocardium. Scale bar = 2 μm.

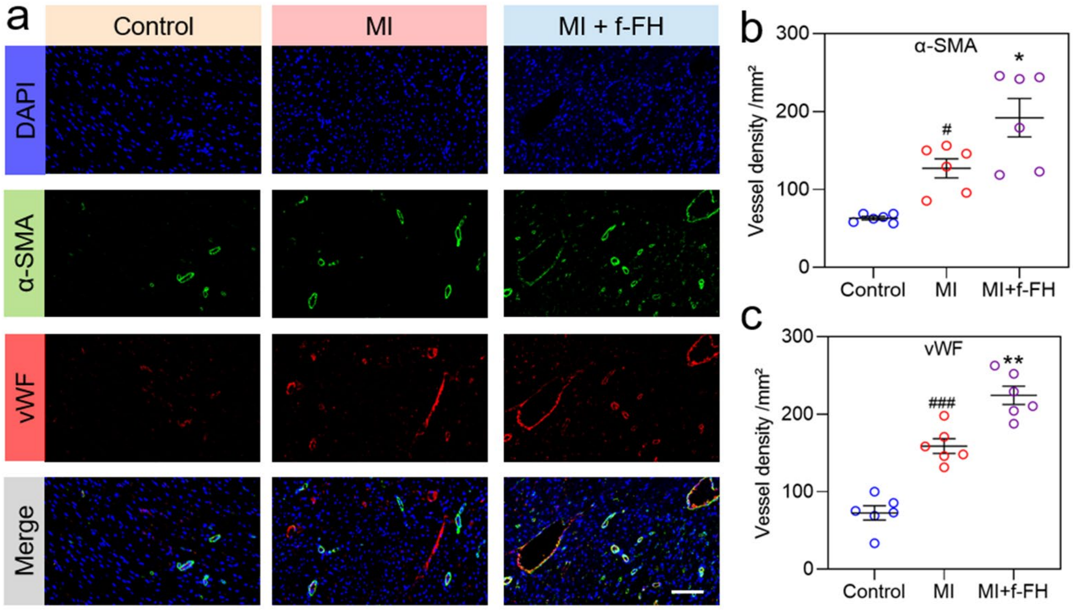

Supplementary Fig. 15. Analysis of neovascularization in myocardium. a Representative image of myocardial vWF/α-SMA immunofluorescence double-staining. Scale bar = 50 μm. **b-c** Quantitative analysis of vWF/α-SMA-positive vascular density ($n = 6$). Compared to the control group, $^{\#}p < 0.05$, $^{\#\#\#}p < 0.001$; compared to the MI group, $^{*}p < 0.05$, $^{**}p < 0.01$.

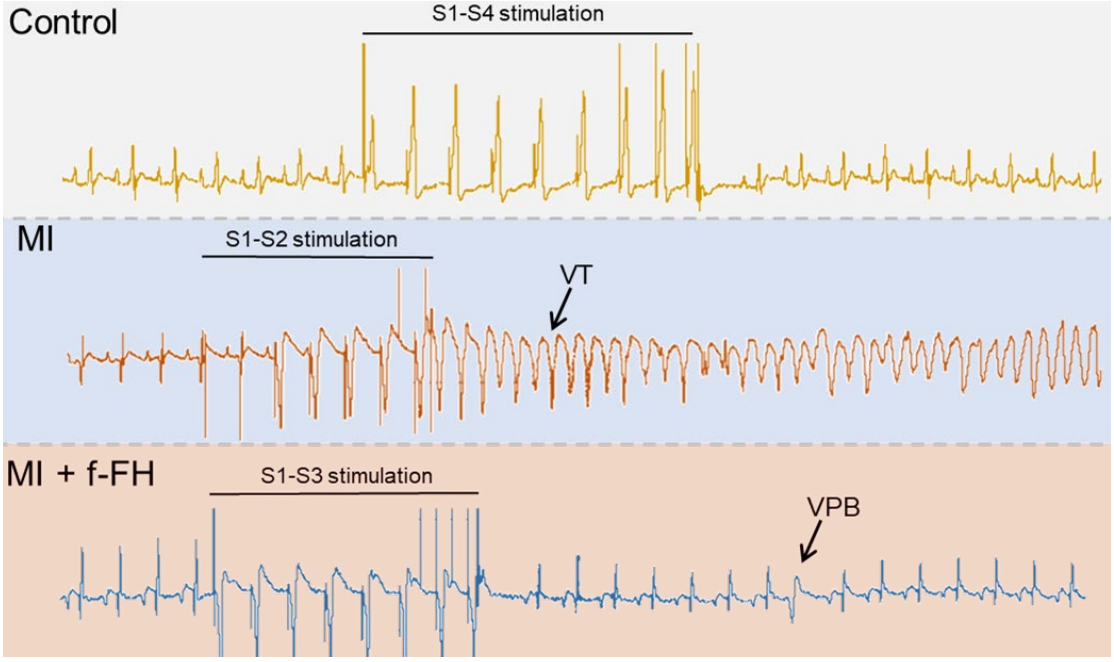

Supplementary Fig. 16. Representative electrocardiograms of ventricular arrhythmia induced by protocol electrophysiological stimulation. VT, ventricular tachycardia. VPB, ventricular premature beat.

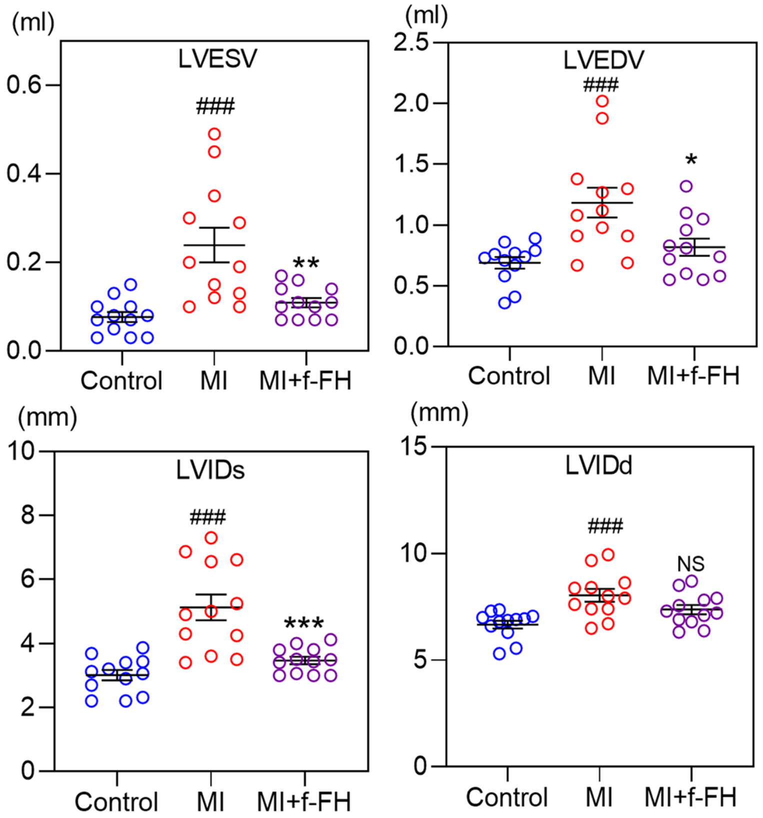

Supplementary Fig. 17. The cardiac function parameters in the three groups. Statistical analysis of LVESV (left ventricular end-systolic volume), LVEDV (left ventricular end-diastolic volume), LVIDs (internal diameter at end-systole), and LVIDd (internal diameter at end-diastole) ($n = 12$). Compared to the control group, $^{###}p < 0.001$; compared to the MI group, $^{*}p < 0.05$, $^{**}p < 0.01$, $^{***}p < 0.001$; NS, no significance.

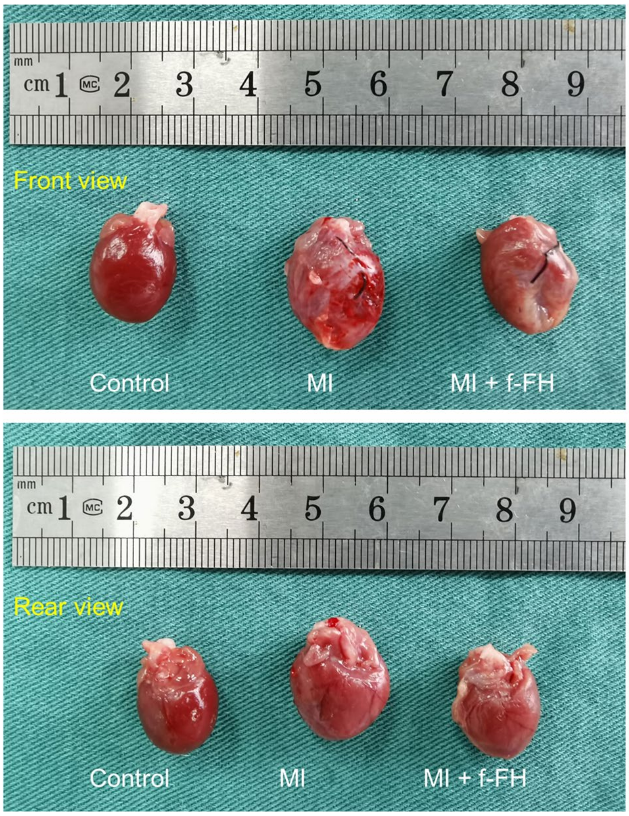

Supplementary Fig. 18. The heart specimens in the control, MI, and MI+f-FH groups.

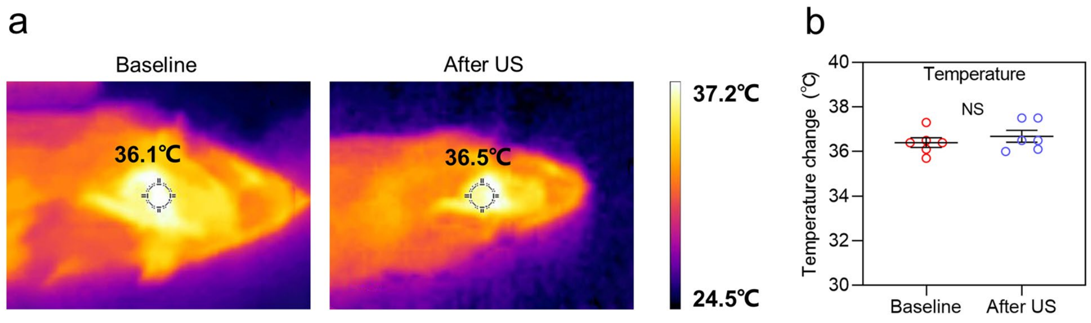

Supplementary Fig. 19. Temperature changes in surrounding tissue of the PVN induced by the f-FH. a Representative image of thermal imaging around the PVN. **b** Statistical analysis of temperature changes (n = 6). NS, no significance.

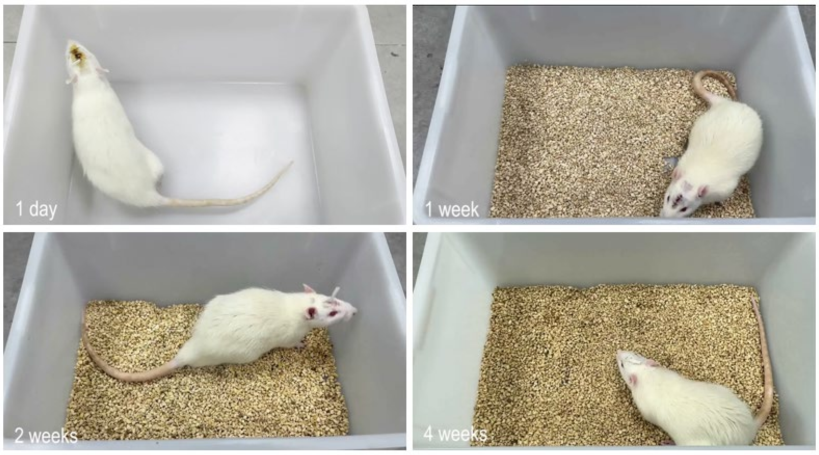

Supplementary Fig. 20. The behavioral observation following the implantation of the f-FH over time.

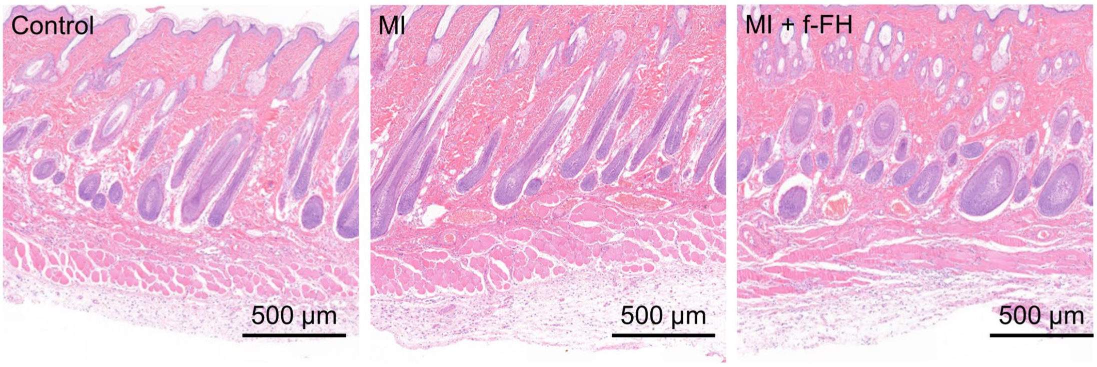

Supplementary Fig. 21. Representative HE staining of skin tissue in the control, MI, and MI+f-FH groups.

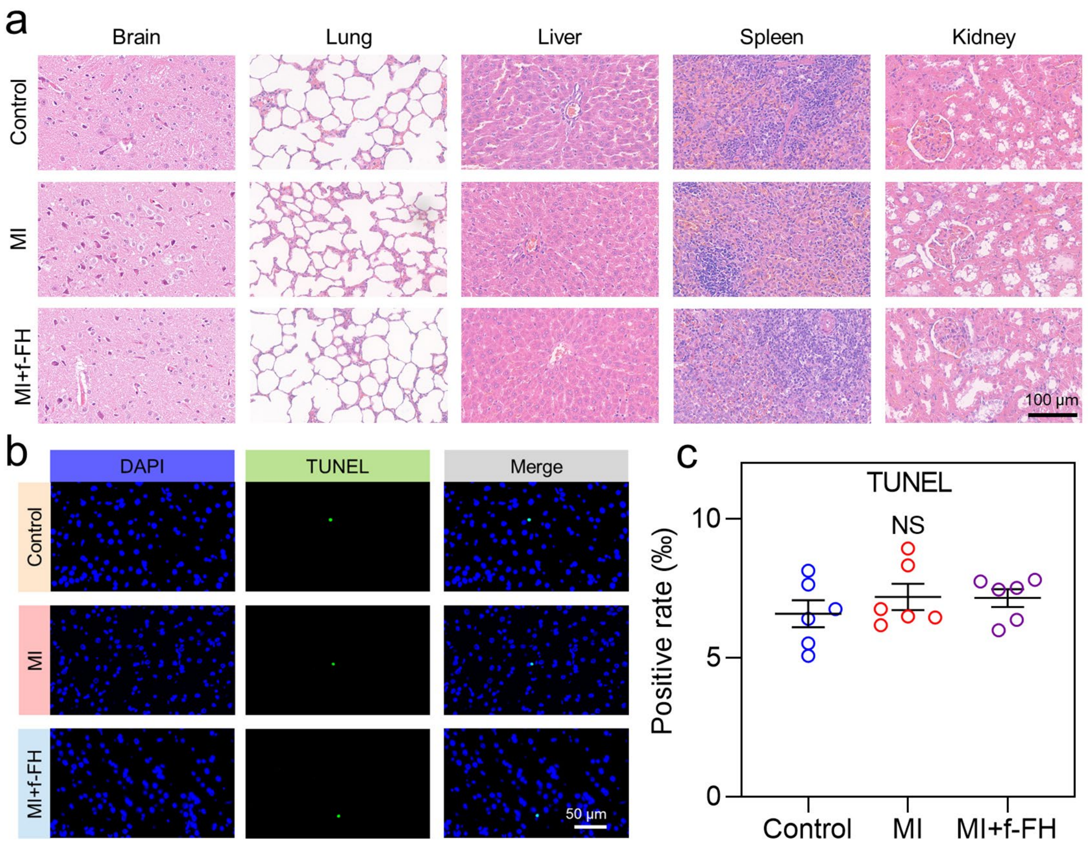

Supplementary Fig. 22. Histological examination of major organs following the PVN ultrasound stimulation. a HE staining in the brain, lungs, liver, spleen, and kidneys. **b** Schematic representation of TUNEL staining in the PVN. **c** Statistical analysis of TUNEL-positive rates in different groups (*n* = 6, respectively). NS, no significance.

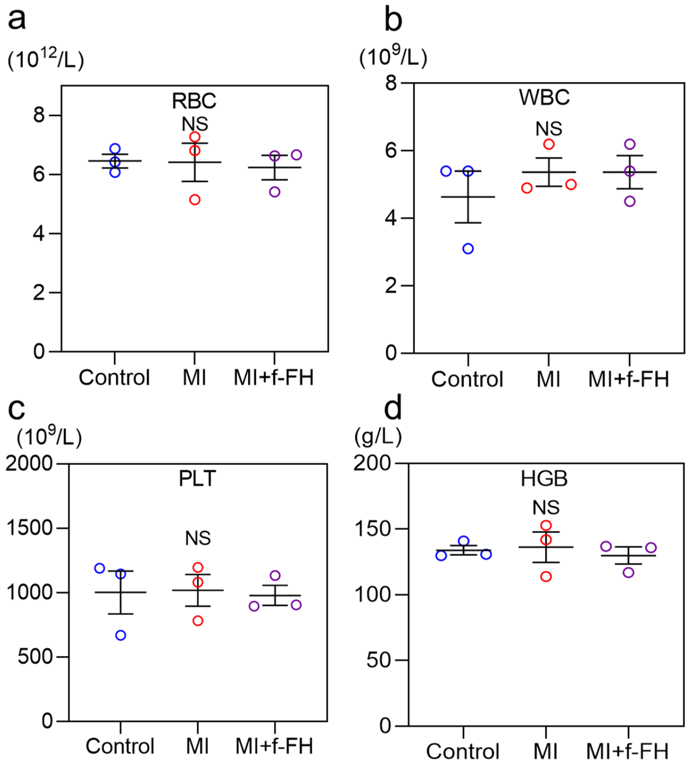

Supplementary Fig. 23. Statistical analysis of blood routine parameters in three groups (*n* = 3, respectively). **a** Red blood cells (RBC). **b** White blood cells (WBC). **c** Platelets (PLT). **d** Hemoglobin (HGB). NS, no significance.

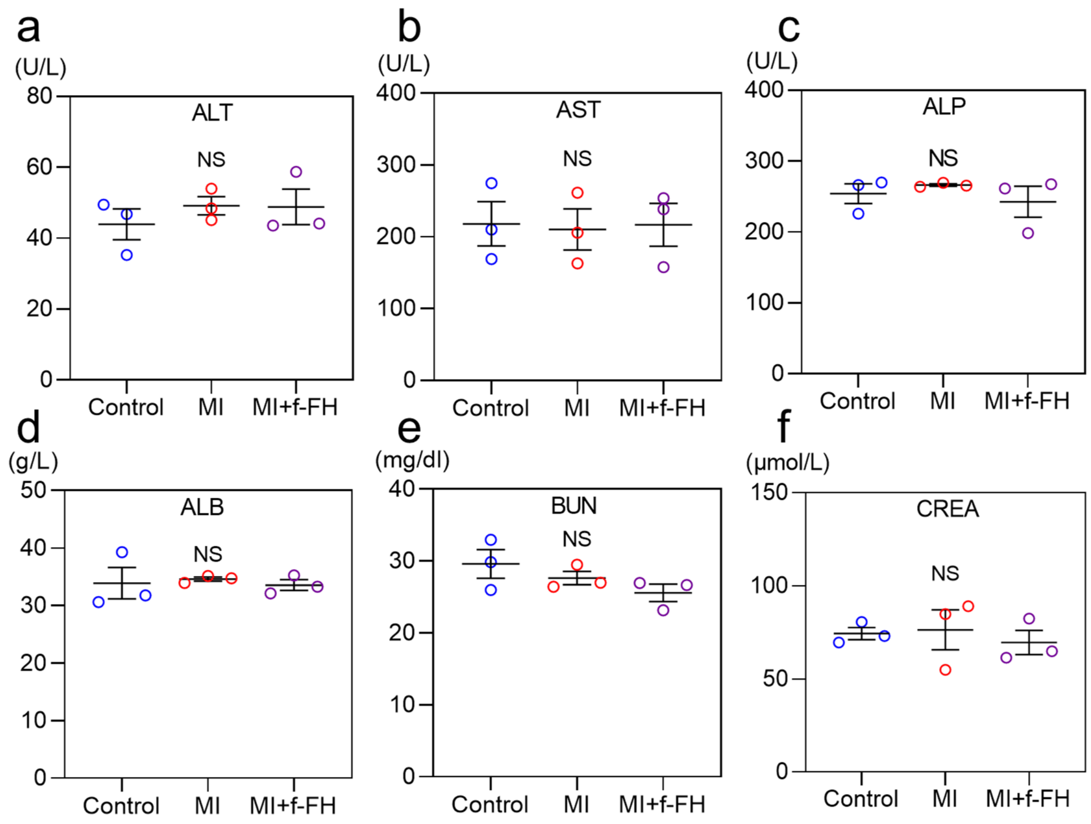

Supplementary Fig. 24. The changes of liver and kidney function in three groups (*n* = 3, respectively). a Alanine aminotransferase (ALT). **b** Aspartate aminotransferase (AST). **c** Alkaline phosphatase (ALP). **d** Albumin (ALB). **e** Blood urea nitrogen (BUN). **f** Creatinine (CREA). NS, no significance.

Supplementary Table 1. Parameters of KNN piezoelectric composites

**Parameters**	**Value**
Center frequency	3.0 MHz
Thickness h	0.7 mm
Ceramic column volume fraction	82%
Piezoelectric coefficient d_{33}	680 pC/N
Electromechanical coupling coefficient k_t	59.2%
Density ρ	4500 kg/m^3
Acoustic velocity c_p	3150 m/s
Acoustic impedance Z_a	14.2 MRayls

Supplementary Table 2. Parameters of the f-FH stimulation for the MI treatment

Parameter	Value
Frequency	3.0 MHz
Sound pressure amplitude	360 kPa
Spatial-peak temporal average intensity of MI treatment (I_{MI})	270.6 mW/cm²
Mechanical index (MI)	0.2
Pulse repetition frequency (PRF)	100 Hz
Duty cycle (DC)	20%
Duration	15 min/d
Interval stimulation strategy	5-s stimulation followed by a 10-s pause
