# Supplementary material for: KNN-based frequency-adjustable ferroelectric heterojunction and biomedical applications
Source: Nat Commun. 2025 Aug 2;16:7120. doi: 10.1038/s41467-025-62079-0 (PMC12317977; doi:10.1038/s41467-025-62079-0)
Supplement: Supplementary file 2 — Description of Additional Supplementary Files [file 41467_2025_62079_MOESM2_ESM.pdf]

Descripfion of Addifional Supplementary Files

Supplementary Movie 1. Transcranial ultrasound stimulation of the PVN based on the f-FH.

Supplementary Movie 2. Biocompatibility study based on the f-FH.
